# Enhancing type 2 diabetes mellitus prediction by integrating metabolomics and tree-based boosting approaches

**DOI:** 10.3389/fendo.2024.1444282

**Published:** 2024-11-11

**Authors:** Ahmet Kadir Arslan, Fatma Hilal Yagin, Abdulmohsen Algarni, Erol Karaaslan, Fahaid Al-Hashem, Luca Paolo Ardigò

**Affiliations:** ^1^ Department of Biostatistics and Medical Informatics, Faculty of Medicine, Inonu University, Malatya, Türkiye; ^2^ Central Labs, King Khalid University, Abha, Saudi Arabia; ^3^ Department of Anesthesiology and Reanimation, Faculty of Medicine, Inonu University, Malatya, Türkiye; ^4^ Department of Physiology, College of Medicine, King Khalid University, Abha, Saudi Arabia; ^5^ Department of Teacher Education, NLA University College, Oslo, Norway

**Keywords:** type 2 diabetes, metabolomics, machine learning, explainable artificial intelligence, biomarkers, predictive modeling

## Abstract

**Background:**

Type 2 diabetes mellitus (T2DM) is a global health problem characterized by insulin resistance and hyperglycemia. Early detection and accurate prediction of T2DM is crucial for effective management and prevention. This study explores the integration of machine learning (ML) and explainable artificial intelligence (XAI) approaches based on metabolomics panel data to identify biomarkers and develop predictive models for T2DM.

**Methods:**

Metabolomics data from T2DM (n = 31) and healthy controls (n = 34) were analyzed for biomarker discovery (mostly amino acids, fatty acids, and purines) and T2DM prediction. Feature selection was performed using the least absolute shrinkage and selection operator (LASSO) regression to enhance the model’s accuracy and interpretability. Advanced three tree-based ML algorithms (KTBoost: Kernel-Tree Boosting; XGBoost: eXtreme Gradient Boosting; NGBoost: Natural Gradient Boosting) were employed to predict T2DM using these biomarkers. The SHapley Additive exPlanations (SHAP) method was used to explain the effects of metabolomics biomarkers on the prediction of the model.

**Results:**

The study identified multiple metabolites associated with T2DM, where LASSO feature selection highlighted important biomarkers. KTBoost [Accuracy: 0.938; CI: (0.880-0.997), Sensitivity: 0.971; CI: (0.847-0.999), Area under the Curve (AUC): 0.965; CI: (0.937-0.994)] demonstrated its effectiveness in using complex metabolomics data for T2DM prediction and achieved better performance than other models. According to KTBoost’s SHAP, high levels of phenylactate (pla) and taurine metabolites, as well as low concentrations of cysteine, laspartate, and lcysteate, are strongly associated with the presence of T2DM.

**Conclusion:**

The integration of metabolomics profiling and XAI offers a promising approach to predicting T2DM. The use of tree-based algorithms, in particular KTBoost, provides a robust framework for analyzing complex datasets and improves the prediction accuracy of T2DM onset. Future research should focus on validating these biomarkers and models in larger, more diverse populations to solidify their clinical utility.

## 1 Introduction

Type 2 diabetes (T2DM), a prevalent metabolic disorder, is characterized by persistent hyperglycemia (1). This condition arises from a deficiency or impairment in insulin secretion, rendering it insufficient to maintain the body’s physiological functions (2). Numerous conventional risk factors for diabetes have been identified, including fasting blood glucose, lifestyle choices, and obesity (3, 4). However, identifying abnormalities in these customary indicators often signifies the presence of diabetes for an extended duration. Consequently, elucidating the metabolic pathways and biomarkers reflecting early changes is crucial for understanding the etiology of T2DM. This knowledge establishes a theoretical foundation for early diagnosis, risk prediction, and the formulation of preventative strategies for T2DM.

In recent decades, numerous innovative technologies have been continually proposed, significantly altering traditional screening strategies. Metabolomics studies and machine learning (ML) algorithms are two prominent technologies used for identifying potential biomarkers and automating the detection or classification of T2DM. Metabolomics offers a comprehensive and systematic analysis of a spectrum of metabolites throughout the disease development process. It holds considerable advantages in revealing abnormal regions, understanding disease occurrence, developmental mechanisms, and facilitating early recognition. This approach not only deepens our comprehension of the biochemical dynamics during disease progression but also enhances the precision of early detection methods (5–9).

However, metabolomics data pose challenges due to their complexity and high dimensionality, making traditional data preprocessing methods and statistical analysis cumbersome. ML algorithms are acknowledged for their effectiveness in handling diverse, large datasets, enabling model training and decision-making based on specific performance indicators. ML has become pivotal in deciphering metabolomics data, showcasing its ability to identify intricate patterns within high-dimensional and heterogeneous datasets (10). The integration of ML techniques with metabolomics has therefore emerged as a vital tool in the rigorous analysis and interpretation of these vast and complex data sets.

By combining numerous weak learners, which individually do not categorize data adequately but perform somewhat better than random prediction, boosting is an ensemble learning strategy that seeks to develop a stronger prediction model. The system achieves this by combining several weak learners. Several boosting-based algorithms are available for both broad and specific applications. A total of three boosting-based prediction models were taken into consideration for this investigation. The integration of metabolomics, boosting-based ML, and explainable artificial intelligence (XAI) has significant promise and advantages for the prediction of T2DM. The use of the Kernel-Tree Boosting (KTBoost) model to identify significant biomarkers may enhance early diagnosis and facilitate personalized treatment strategies for T2DM. The algorithm demonstrates proficiency in managing complex metabolomics datasets and has the potential to improve T2DM prevention and management in practical clinical environments by effectively integrating these advanced methodologies (11).

An exhaustive exploration of metabolomics technology in T2DM has revealed that metabolic markers from diverse regulatory pathways, including sugar, fat, and protein regulation, exhibit intimate associations with the onset and progression of T2DM (12–14). Numerous studies have conducted metabolomics analyses to identify biomarkers linked to T2DM (15–18). However, due to the abundance of metabolites, outcomes have exhibited variations across different studies. Hence, there is a necessity to distinguish and integrate existing metabolic biomarkers for T2DM. This requirement underscores the importance of a more refined selection and validation process that can leverage advanced computational tools to improve the robustness and reproducibility of findings.

Based on the comprehensive biomarker discovery process, the current research aims to identify distinctive biomarkers associated with T2DM and create an optimal prognostic model that can accurately predict the likelihood of T2DM occurrence in patients.

## 2 Materials and methods

### 2.1 Data collection

The NIH Common Fund National Metabolomics Data Repository (NMDR) at Metabolomics Workbench (www.metabolomicsworkbench.org) provided the data utilized in this investigation. The data were accessible under project ID ST002681. The Inonu University Health Sciences Non-Interventional Clinical Research Ethics Committee approved this study (approval number: 2024/5862). Using MetSizeR and the PPCA model as a basis, the sample size needed for this investigation was computed by setting the false discovery rate to 0.05. This led to the estimation of a minimal sample size of 14 patients overall, with 7 individuals in each category. T2DM screening followed the American Diabetes Association Standards of Medical Care standards by dividing patients into control (*n* = 34) or T2DM (*n* = 31) groups. Smoking status in all participants and pregnancy and contraceptive use in female participants were the exclusion criteria for the study. This study utilized metabolomics data consisting of a variety of metabolites. The majority of metabolites belonged to the following classes: amino acids and peptides (41), fatty esters (38), fatty acids (20), and purines (17) (19).

### 2.2 Methods

**Figure 1:**
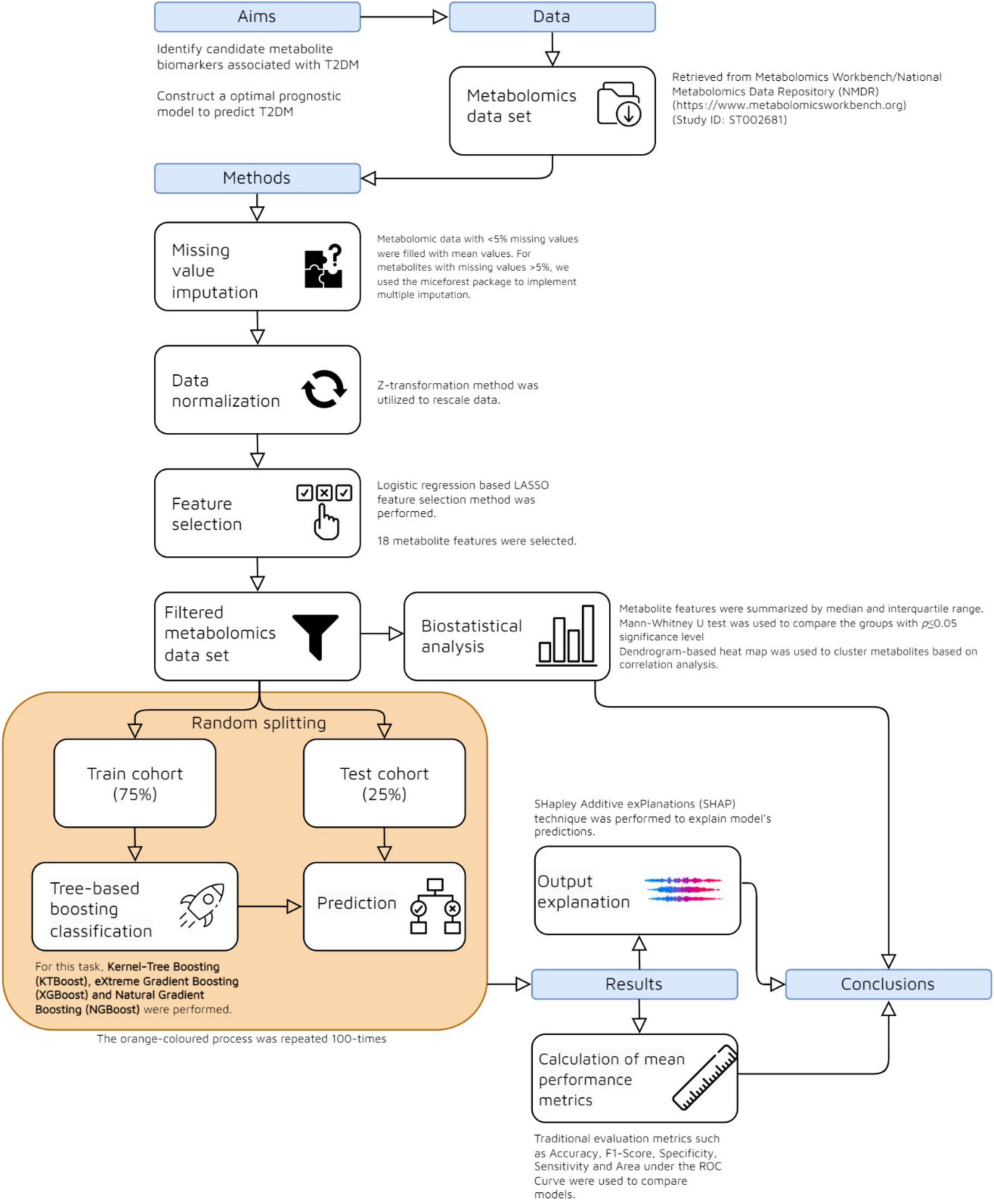
Flow chart of the methodology for interpretable prediction of T2DM.

This article’s focus is on the identification and categorization of T2DM biomarkers using the metabolomics dataset mentioned in the preceding section. Two primary phases comprise the machine learning process for producing explainable classifiers: (i) building and assessing various tree-based learning models and (ii) using the SHapley Additive exPlanations (SHAP) algorithm to understand global outputs. The flow-chart summarizing the methodology used in the study is presented in **Figure 1**.

### 2.3 Data preprocessing

Metabolomics data with<5% missing values were filled with mean values. For metabolites with missing values >5%, we used the miceforest package to implement multiple imputations. To increase the stability of the models, all continuous variables were normalized to obtain a distribution with a mean of 0 and a standard deviation of 1 (20, 21).

### 2.4 Feature selection

Regression analysis employs the Least Absolute Shrinkage and Selection Operator (LASSO) as a regularization technique, incorporating an L1 norm penalty term into the objective function of standard regression models. In the context of binary classification problems, LASSO introduces an L1-norm penalty component to the negative log-likelihood function within logistic regression. This aims to estimate regression coefficients through minimization. After LASSO regression analysis, the model conducts feature selection by examining the coefficients assigned to each predictor variable. Due the L1 norm penalty, which encourages sparsity in the coefficients, shrunk some coefficients to zero, effectively eliminating the corresponding predictors from the model. final model retains features with non-zero coefficients, considering them important for prediction, and excludes those with zero coefficients as less influential (22–24). The regularization parameter value for LASSO was accepted by default (1.0).

### 2.5 Construction and assessment of prediction models

Based on metabolomics data, three tree-based ML models were built to predict T2DM. During the modeling phase, the KTBoost method—which combines the tree and kernel approaches—was employed in addition to the tree-based XGBoost and NGBoost approaches, which have limited interpretability. These algorithms were selected primarily because of their ability to cope with missing data rapidly, prevent overfitting, and achieve high prediction power (11, 22–24). The 65 patients were split 4:1 into training and testing groups using a stratified random sample technique. Ultimately, an assessment and comparison of each model’s performance on the test set were conducted. We performed the persistence technique 100 times using various random seeds and determined the average performance over these 100 times to achieve a more reliable performance estimate, prevent reporting biased findings, and reduce overfitting. Area under the Curve (AUC), F1 score, specificity, accuracy, and sensitivity criteria were applied to assess the models’ performance. After a thorough analysis of several performance factors, the best-performing model out of the three models used for the categorization was chosen for global explanation.

### 2.6 Machine learning algorithms

This study investigated three tree-based ensemble ML algorithms for T2DM prediction: KTBoost, XGBoost, and NGBoost. These algorithms are all variations of gradient boosting, which iteratively builds decision trees on subsets of the data, aiming to improve the model’s performance with each new tree. These approaches were chosen because these algorithms effectively handle high-dimensional data and the results are reliable, interpretable, and applicable to examining complex relationships.

#### 2.6.1 Kernel-Tree Boosting

Boosting algorithms are frequently utilized in both ML and data science for enhancing predictive performance on intricate datasets by iteratively amalgamating weak classifiers to diminish both bias and variance. Unlike typical approaches where a single type of function forms the basis of learners, the KTBoost algorithm uniquely integrates either a regression tree or a penalized Reproducing Kernel Hilbert Space (RKHS) into its ensemble of base classifiers. During each iteration of the boosting process, denoted by q, KTBoost employs a candidate tree [ $f_{q}^{T}(x)$ ] and an RKHS function [ $f_{q}^{K}(x)$ ] as strategies for minimizing based on a second-order Taylor approximation of the form ${\text{Ω}}^{2}(\psi_{q}+f)$ utilizing Newtonian or gradient-based optimization techniques. The base learner that achieves the minimum empirical risk Ω is selected for inclusion in the ensemble. This selection process, whether it opts for the tree or the RKHS function, is determined by which addition results in the lowest overall risk, adhering to the relationship shown in equation (1):


$$\left(x)=\psi_{q-1}(x)+v\times f_{q}(x\right)$$

where *v* represents the shrinkage factors, crucial for updating the model to enhance function understanding across varying degrees of regularity, encapsulating both discontinuous and smooth elements. Typically, discontinuities are addressed using regression trees, while continuous or smooth aspects are managed via RKHS functions (11, 25).

#### 2.6.2 eXtreme Gradient Boosting

XGBoost is another powerful tree-boosting algorithm known for its efficiency, regularization techniques to prevent overfitting, and scalability for handling large datasets (26). These features make XGBoost a strong contender for T2DM prediction tasks.

#### 2.6.3 Natural Gradient Boosting

NGBoost is a tree-boosting algorithm specifically designed for analyzing ecological and environmental data. While not as widely used for general machine learning tasks, NGBoost can be particularly useful if your metabolomics data contains features related to dietary or environmental factors potentially influencing T2DM risk (24).

### 2.7 Performance evaluation metrics

Several metrics were employed to evaluate the performance of the ML models for T2DM prediction (27):

- Accuracy: Measures the overall proportion of correct predictions made by the model. This is calculated as the ratio of true positives and true negatives to the total number of cases.
- F1-Score: Represents the harmonic mean of precision and recall, incorporating both measures into a single score.
- Sensitivity (Recall): Measures the proportion of true positives correctly identified by the model (true positive rate).
- Specificity: Measures the proportion of true negatives correctly identified by the model (true negative rate).
- Area under the Curve (AUC): Represents the area under the receiver operating characteristic (ROC) curve. This metric assesses the model’s ability to discriminate between cases with and without T2DM.

### 2.8 Interpretable modeling and the importance of metabolites

ML models are sometimes called “black boxes” because it can be challenging to comprehend the reasoning behind an algorithm’s ability to provide accurate predictions for a certain patient population (28, 29). Therefore, global explanations of black box ML models were obtained in this work using the SHAP approach. Prioritizing the features in the final model based on their importance helped identify important T2DM biomarkers in the patient group using the SHAP technique.

#### 2.8.1 SHapley Additive exPlanations

SHAP is a method in the field of ML that describes the output of any model by calculating the contribution of each feature to the prediction. It is based on Shapley values, a concept from cooperative game theory that distributes total winnings among players according to their marginal contribution to the overall success of the group. SHAP can be applied to any ML model and provides a consistent method for interpreting results across different algorithms. It breaks down a prediction to show how much each feature contributes positively or negatively to the target variable. While SHAP values can explain individual predictions, aggregating SHAP values into a dataset provides insights into the overall behavior of the model and highlights which features are most important globally (30–32).

### 2.9 Biostatistical analysis

The interquartile range (IQR) and median are used to summarize quantitative data. The Shapiro-Wilk test was used to examine the normal distribution. The Mann-Whitney U test was used to determine if there was a statistically significant difference in the input factors and the connection between the categories of the output variable, the “control” and “T2DM” groups. A Heatmap graph based on the Spearman rho coefficient was drawn to examine the relationships between metabolite levels. A p ≤ 0.05 was deemed statistically significant. IBM SPSS Statistics for Windows version 28.0 (New York, USA) was used for all statistical analyses.

## 3 Results

At first, there were 178 metabolite features, and the LASSO was used to find important metabolite biomarkers in T2DM. These biomarkers were found to be high-dimensional for making clinical prediction models easier to understand and more reliable. After LASSO feature selection, Laspartate, lcysteine, llysine, lcystine, adenine, xanthine, dglucosamine, creatine, l1pyrroline3hydroxy5carboxylate, taurine, 3sulfinolalanine, lcysteate, serotonin, tiglylcarnitine, dimethylnonoylcarnitine, octadecenylcarnitine, phenyllactate(pla), and riboflavin metabolites were identified as important biomarkers in T2DM. **Table 1** contains the statistics results regarding the changes in these biomarkers between T2DM and controls. **Table 1** compares metabolite levels between control subjects and T2DM subjects using medians and IQRs for each group. It also includes *p* values for statistical tests comparing two groups. Lcysteine metabolite levels were significantly lower (*p* = 0.002) in T2DM (Median: 0.005, IQR: 0.009) compared to controls (Median: 0.011, IQR: 0.012). Phenylactatepla was significantly higher (*p*=0.007) in T2DM (Median: 0.006, IQR: 0.008) compared to control (Median: 0, IQR: 0.006). In addition, the median concentration of laspartate is lower in the T2DM group compared to the control group (*p*=0.048).

**Table 1:** Comprehensive results of univariate statistical analysis.

Metabolite	Control	T2DM	p
	Median (IQR)	Median (IQR)	
**laspartate**	0.029 (0.04)	0.013 (0.019)	**0.048**
**lcysteine**	0.011 (0.012)	0.005 (0.009)	**0.002**
**llysine**	0.225 (0.174)	0.231 (0.145)	0.984
**lcystine**	0.006 (0.005)	0.003 (0.008)	0.258
**adenine**	0.004 (0.005)	0.002 (0.003)	0.073
**xanthine**	0.007 (0.008)	0.007 (0.005)	0.953
**dglucosamine**	0.002 (0.004)	0.002 (0.007)	0.783
**creatine**	0.966 (0.049)	0.961 (0.04)	0.964
**l1pyrroline3hydroxy5carboxylate**	0.081 (0.078)	0.089 (0.071)	0.281
**taurine**	0.009 (0.016)	0.012 (0.018)	0.510
**3sulfinolalanine**	0 (0.001)	0 (0)	0.886
**lcysteate**	0.004 (0.004)	0.004 (0.004)	0.167
**serotonin**	0.001 (0.001)	0.001 (0.001)	0.293
**tiglylcarnitine**	0.003 (0.002)	0.003 (0.002)	0.188
**dimethylnonoylcarnitine**	0.001 (0.001)	0 (0)	0.080
**octadecenylcarnitine**	0.003 (0.006)	0.003 (0.009)	0.860
**phenyllactatepla**	0 (0.006)	0.006 (0.008)	**0.007**
**riboflavin**	0.001 (0.002)	0.002 (0.004)	0.678

T2DM, type 2 diabetes mellitus; IQR, interquartile range.

P values ​​less than the statistical significance level (0.05) are indicated in bold.

**Figure 2:**
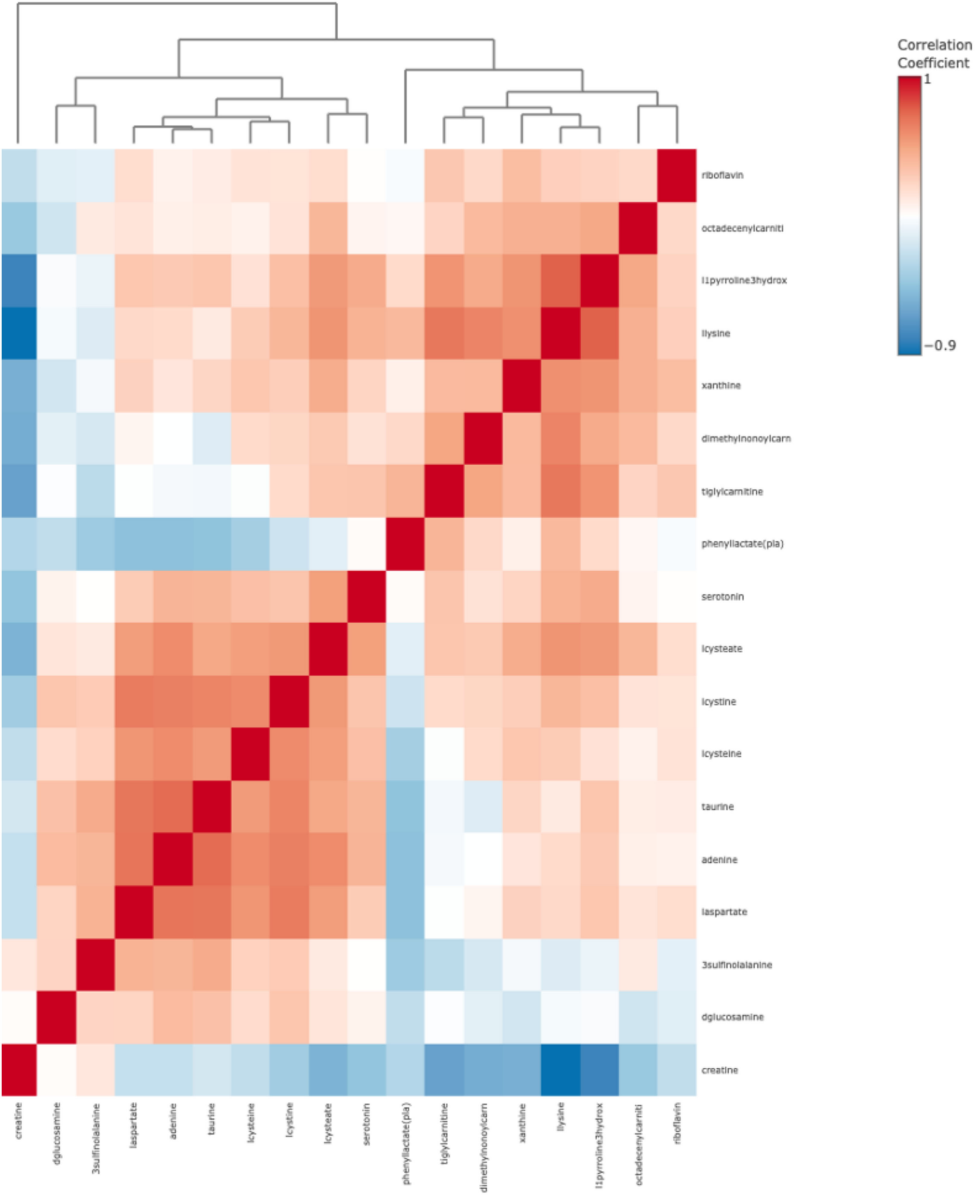
Heat map for metabolite biomarkers identified after LASSO in T2DM.

In **Figure 2**, the heat map displays correlation coefficients ranging from -1 (strong negative correlation, represented in blue) to +1 (strong positive correlation, represented in red). Dendrograms (tree-like structures at the top and left of the heat map) group metabolites based on the similarity of their correlation profiles; this may be useful in identifying potential biomarkers or metabolic signatures specific to T2DM or controls. A positive correlation (solid red squares) was observed between metabolites such as riboflavin, octadecenylcarnitine, and 1lproline3hydroxy. Based on this, it means that these metabolites may increase and decrease together in the metabolic profiles of patients. These results may suggest regulatory mechanisms to clinicians in patients. Negative correlations indicate that certain compounds may affect each other in opposite directions, reflecting mutual regulatory effects that may play a role in metabolic homeostasis. A negative correlation was observed between creatine and l1pyroline3hydroxy5carboxylate. The results indicate that an increase in creatine levels may be associated with a decrease in l1pyroline3hydroxy5carboxylate levels.

**Table 2** compares the performance metrics of three predictive models (KTBoost, XGBoost, and NGBoost) in T2DM classification using the 100 repeated hold-out method and mean values for Accuracy, F1 Score, Sensitivity, Specificity, and AUC measurements along with 95% CIs are given for each model. KTBoost achieved the highest Accuracy (0.938) and F1 Score (0.943) among the three models in detecting T2DM; as a result, it can be said that there is a strong balance between sensitivity and specificity of the model. The model also has the highest Sensitivity value (0.971), resulting in the KTBoost model having the best performance in detecting positive cases (T2DM) compared to related models. When the specificity (0.903) and AUC (0.965) values were examined, it was determined that the model had a very strong general discrimination ability. Accuracy (0.908), AUC (0.945), and F1 Score (0.914) of the XGBoost model are slightly lower than KTBoost, and it can be stated that the model performance is good but slightly lower than KTBoost. NGBoost has the lowest Accuracy (0.892) and F1 Score (0.899) among the three models. The sensitivity (0.939) value is comparable to XGBoost, showing a perfect true positive rate. NGBoost has the lowest Specificity (0.844), indicating that it is less powerful than other models in accurately detecting negative cases (control).

**Table 2:** T2DM prediction performance metrics of the constructed models.

Metric/Model	KTBoost	XGBoost	NGBoost
**Accuracy**	0.938 (0.880-0.997) SD: 0.029	0.908 (0.837-0.978) SD: 0.036	0.892 (0.817-0.968) SD: 0.038
**F1-Score**	0.943 (0.886-0.999) SD: 0.028	0.914 (0.846-0.982) SD: 0.034	0.899 (0.825-0.972) SD: 0.037
**Sensitivity**	0.971 (0.847-0.999) SD: 0.038	0.941 (0.803-0.993) SD: 0.048	0.939 (0.798-0.993) SD: 0.049
**Specificity**	0.903 (0.742-0.980) SD: 0.060	0.871 (0.702-0.964) SD: 0.066	0.844 (0.672-0.947) SD: 0.070
**AUC**	0.965 (0.937-0.994) SD: 0.014	0.945 (0.906-0.984) SD: 0.019	0.936 (0.891-0.980) SD: 0.022

AUC, Area under the Curve; KTBoost, Kernel-Tree Boosting; XGBoost, eXtreme Gradient Boosting; NGBoost, Natural Gradient Boosting; SD, standard deviation.

**Figure 3:**
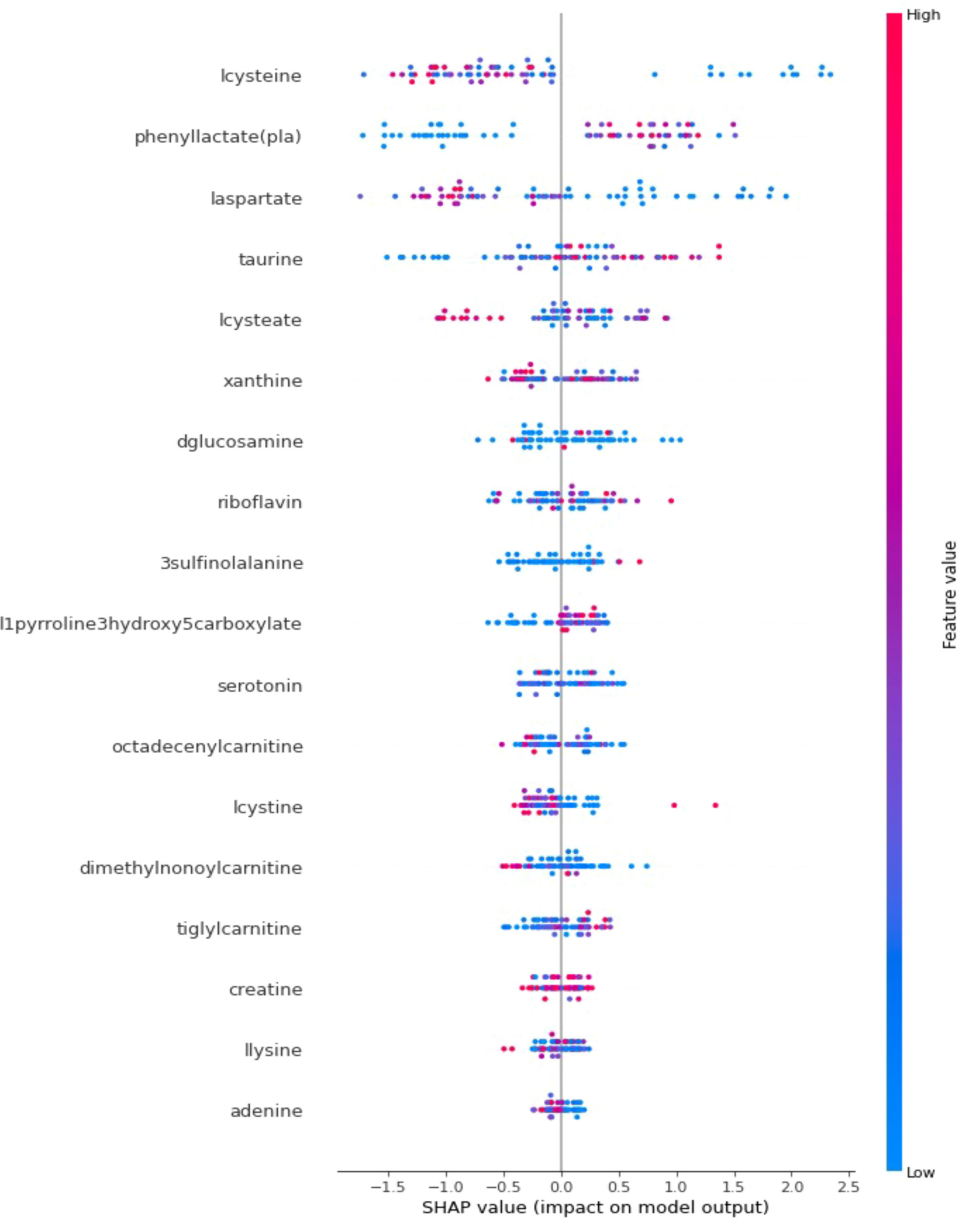
SHAP explanations for the optimal model KTBoost; X-axis: Represents SHAP values that measure the impact of each metabolite on the model’s output. Positive SHAP values increase the probability that the model predicts T2DM, while negative values decrease this probability; Y-axis: Lists metabolites in order of importance by impact on model output; Red indicates high values of the corresponding metabolite and blue indicates low values. The color intensity and position on the X-axis express how effective the level of the metabolite is in predicting diabetes.

**Figure 4:**
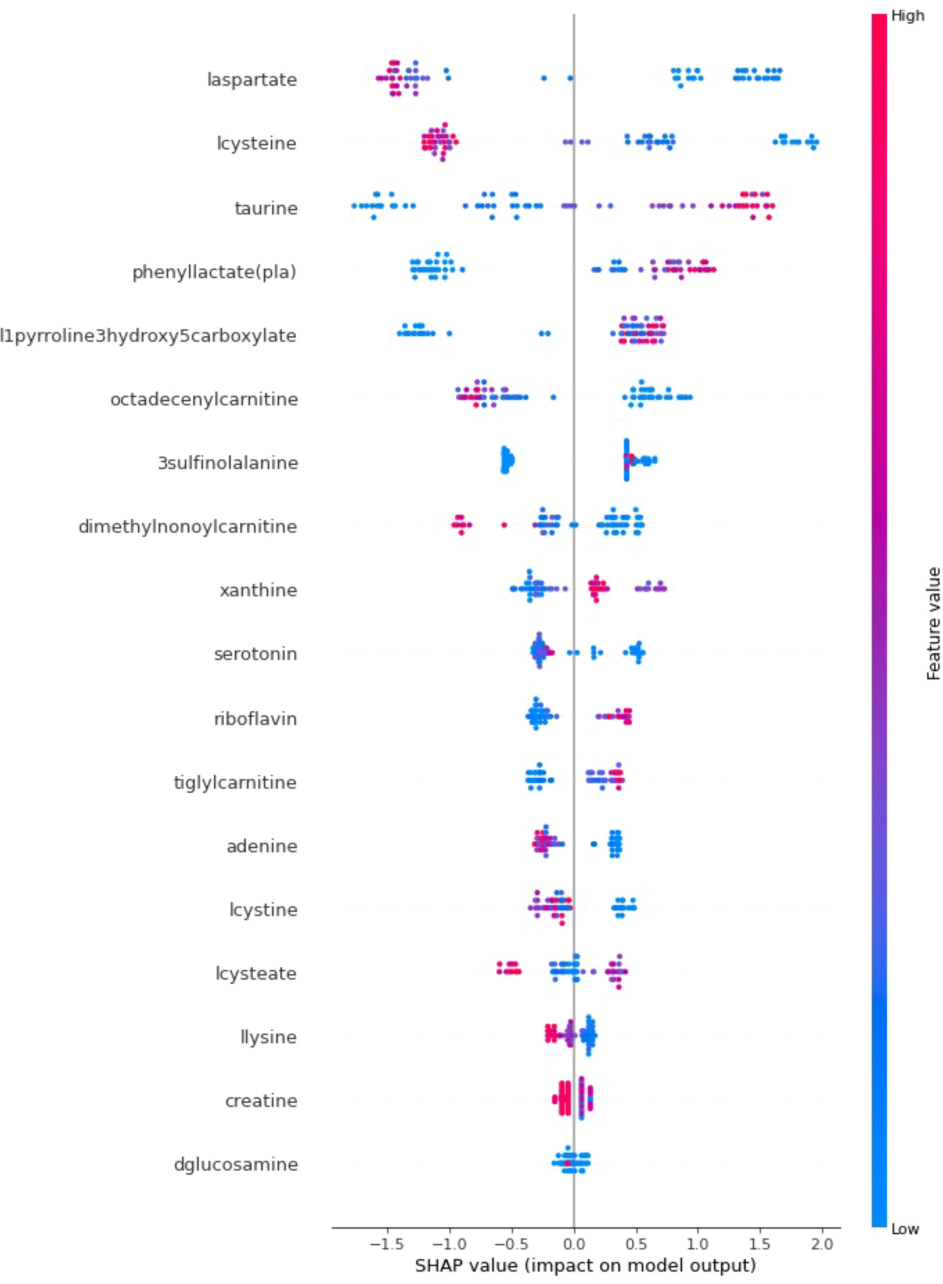
SHAP explanations for the optimal model XGBoost.

**Figure 5:**
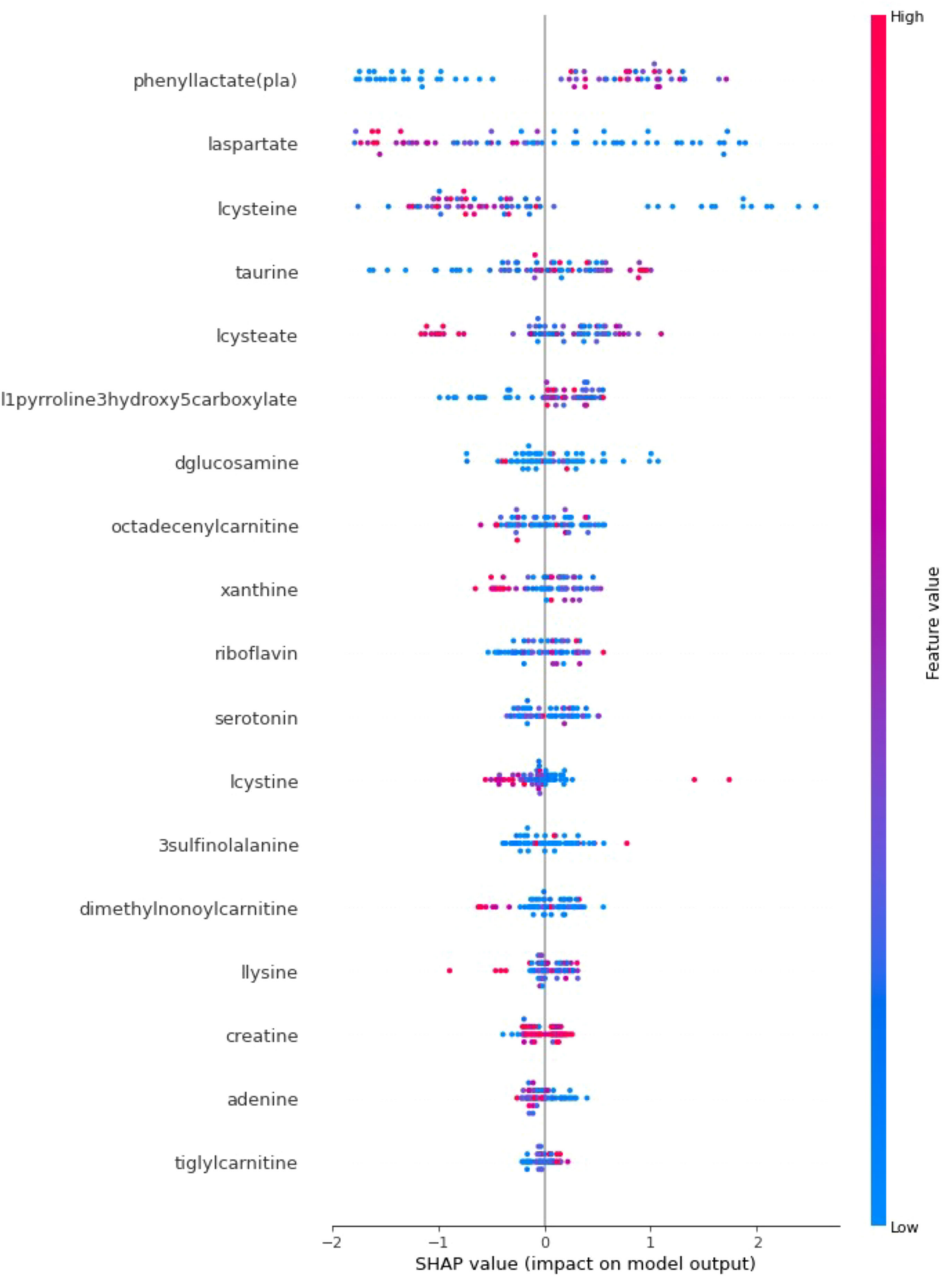
SHAP explanations for the optimal model NGBoost.

In addition to achieving optimal prediction accuracy, it is crucial to evaluate the relative importance of various contributing factors and quantify their impact on prediction results. Therefore, in this section, in addition to the optimal KTBoost model, SHAP analysis was used to interpret the results of the XGBoost and NGBoost models. SHAP graphs for the KTBoost, XGBoost and NGBoost models are presented in **Figures 3**–**5**, respectively. The SHAP summary chart was created to determine the importance of different contributing factors and explain their impact. In this plot, all input factors are listed on the Y-axis, arranged in decreasing order of importance. The importance of a factor refers to the degree to which it affects the output of various factors of a model. The importance of the factor is visually represented by a color gradient from blue to red. In the SHAP plot, the X-axis represents the SHAP value, and the Y-axis represents the names of the features. Shapley values indicate how much each feature affects the prediction of the target variable. Each point on the graph represents the SHAP value of a single sample (patient) relative to a specific metabolite.

When the SHAP explanations for the optimal prediction model, KTBoost, are examined, metabolites such as cysteine, phenyllactate (pla) and laspartate at the top of the plot have more scattered points along the SHAP value spectrum, indicating that they have a significant impact on the prediction model. The metabolites lcysteine, laspartate and lcysteate show a high distribution of positive SHAP values, explaining that lower concentrations of lcysteine, laspartate and lcysteate are strongly associated with the presence of T2DM. In contrast, it is a result of SHAP statements that high levels of phenyllactate (pla) and taurine metabolites increase the risk of T2DM.

## 4 Discussion

This study investigated the potential of a hybrid ML and explainable artificial intelligence (XAI) approach for discovering metabolomic biomarkers and developing a prognostic model for T2DM. The findings demonstrate the effectiveness of this combined strategy for advancing our understanding and prediction of T2DM.

Currently, A1C Test, Fasting Plasma Glucose (FPG) Test, Oral Glucose Tolerance Test (OGTT) and Random Plasma Glucose Test are used for the diagnosis of Type 2 DM. Although these methods have various advantages, they suffer from time, cost, inaccurate results in various clinical cases, false positivity, misleading single measurement, etc. (33–35). At this point, the use of ML models in the diagnosis of T2DM can provide good validation of the above-mentioned routine tests and prevent unnecessary retests. ML algorithms can be effective in early diagnosis of type 2 diabetes mellitus and can provide higher accuracy rates and personalized diagnostic models by working with large data sets. A comprehensive review study (36) asserted that ML models accurately classify Type 2 Diabetes Mellitus with high performance, specifically highlighting the superior prediction performance of decision tree-based models over other models. In addition, an observational study (37) has shown that the Gradient Boosting Machine model, one of the decision tree-based boosting models used to predict T2DM, offers better prediction performance than other classical ML models.

The application of LASSO-based feature selection successfully identified a subset of critical metabolites from the metabolomics data, highlighting the power of this technique in managing high-dimensional datasets (38). Notably, the identified biomarkers, including lcysteine, laspartate, and phenyllactate (pla), are known to be involved in metabolic pathways associated with T2DM (39). This alignment with established metabolic knowledge strengthens the validity of our results and suggests potential mechanisms underlying T2DM development.

The comparative analysis of ML algorithms revealed KTBoost as the optimal model for T2DM prediction. This finding underscores the strengths of gradient boosting frameworks in handling complex, non-linear data often encountered in metabolomics studies (40). Further investigation into the specific hyperparameters tuning strategies employed for the KTBoost model could provide valuable insights into optimizing ML algorithms for metabolomics-based T2DM prediction. Additionally, exploring the performance of the KTBoost model in comparison with other advanced ML algorithms, such as deep learning architectures, could offer valuable insights into the trade-offs between model complexity, interpretability, and generalizability in this context.

The KTBoost model’s calculated SHAP values for metabolite variables show relatively closer intervals, indicating a more balanced effect distribution among the variables. The XGBoost model reveals that certain variables, such as l-cysteine, phenylacetate (pla), and laspartate, exhibit more significant effects than others. In the NGBoost model, a dominant negative effect of the l-cysteine metabolite in particular stands out. Examining the SHAP graphs in **Figures 3**–**5** reveal a negative correlation between the risk of T2DM and the increase in the concentration amounts of the metabolite variables l-cysteine and laspartate in all models. Furthermore, despite differences in their orders and effect sizes across all three models, we can assert that similar metabolite variables such as l-cysteine, phenylacetate (pla), and laspartate significantly influence the predictive performance of the models compared to other variables. The utilization of SHAP values for interpreting the KTBoost model provided valuable clinical insights. The observed association between lower levels of lcysteine and laspartate with T2DM suggests potential metabolic deficiencies in pre-diabetic stages, possibly due to oxidative stress or inflammatory processes (41). Conversely, elevated levels of phenyllactate (pla) and taurine emerged as potential early T2DM risk factors, highlighting their potential utility in preventive strategies. Future research could explore the biological mechanisms underlying these metabolite level changes in the context of T2DM development, potentially through *in vitro* or *in vivo* studies. Investigating the influence of modifiable lifestyle factors, such as diet and exercise, on these metabolite levels could inform the development of personalized interventions for T2DM prevention.

The findings of this study hold significant promise for improving clinical practice in T2DM management. Early detection of T2DM is crucial for preventing or delaying the onset of complications such as neuropathy, nephropathy, and retinopathy (42). The current diagnostic gold standard, HbA1c testing, often identifies individuals only after they have developed hyperglycemia. By identifying potential biomarkers associated with pre-diabetic stages, this approach has the potential to facilitate earlier intervention and potentially prevent the progression to overt T2DM.

The American Diabetes Association (2020) (42) emphasizes the importance of lifestyle modifications, including dietary changes and increased physical activity, as the cornerstone of T2DM management. Integrating metabolomics profiling with these established strategies could enable the development of more personalized interventions. For example, individuals with lower levels of lcysteine or laspartate might benefit from dietary modifications rich in these amino acids, while those with elevated phenyllactate (pla) levels could be targeted with interventions aimed at addressing potential underlying metabolic imbalances.

Metabolomics research has found many metabolites, including branched-chain amino acids, glycine, glucose, fructose, and lipids, that correlate with the risk of T2DM. Particular metabolites such as leucine, alanine, and oleic acid have a positive correlation with T2DM, while lysophosphatidylcholine and creatinine show a negative correlation. Metabolomics may discover new biomarkers that enhance the prediction of T2DM beyond conventional clinical risk factors. ML methods that include metabolomics data may substantially enhance the prediction of T2DM. Models using metabolomics data demonstrated superior prediction ability (AUC = 0.77) in contrast to models relying only on clinical risk variables (AUC = 0.68). Metabolomics-based models may detect early metabolic alterations that predate the clinical manifestation of T2DM, facilitating more effective preventative measures. These results indicate that the incorporation of metabolomics with tree-based boosting methods may significantly improve the prediction of T2DM by discovering new metabolic biomarkers and enhancing predictive accuracy beyond conventional clinical risk variables (36, 43, 44).

While this study offers promising avenues for T2DM diagnosis and management, future research is warranted to further strengthen its foundation. Longitudinal validation of these biomarkers across diverse populations, including different ethnicities and age groups, is crucial to assess their generalizability and reliability. Additionally, integrating these predictive models into clinical workflows needs investigation to evaluate their effectiveness in real-world settings and their potential to improve patient outcomes. This could involve developing user-friendly interfaces for clinicians and exploring cost-effectiveness considerations for incorporating metabolomics profiling into routine clinical practice.

Furthermore, exploring the interplay between these biomarkers and other relevant data points, such as gut microbiota composition and genetic predispositions, could lead to a more comprehensive understanding of T2DM etiology. Integrating metabolomics data with these other domains could potentially lead to the identification of multi-factorial signatures with enhanced diagnostic accuracy and the development of more targeted treatment strategies. Machine learning algorithms capable of handling multi-omics data integration could be particularly valuable in this endeavor. By furthering our understanding of the complex interplay between these factors, we can move towards a more holistic approach to T2DM prevention and treatment.

This study successfully combined metabolomics, ML, and XAI to develop a novel approach for T2DM prediction. The identified biomarkers and the KTBoost model hold promise for early diagnosis and personalized treatment strategies. Future research directions outlined in this discussion pave the way for further refinement and real-world implementation of this innovative approach, ultimately contributing to more effective T2DM prevention and management.

## 5 Limitation

Despite the significant and robust results obtained in the study, there were several limitations. First, the sample size was relatively small, with only 31 T2DM patients and 34 healthy controls. This limited sample size may affect the generalizability of our findings to larger, more diverse populations. Future studies should aim to include a larger and more representative cohort to validate the results and ensure their applicability across different demographics. In addition, the study primarily used metabolomics data, while providing valuable insights into metabolic changes associated with T2DM, may not capture the full complexity of the disease. Integrating additional omics data, such as genomics, proteomics, and transcriptomics, may provide a more comprehensive understanding of T2DM and increase the robustness of predictive models. Subsequent research should consider conducting additional experimental validation, such as targeted metabolomics studies or *in vitro* experiments, to confirm the roles of these biomarkers in T2DM development.

## 6 Conclusion

This study constructed a new interpretable prediction model based on XAI to predict patients with T2DM early. Combining the kernel and tree approach, KTBoost has increased both accuracy and robustness. Furthermore, physicians can comprehend the underlying metabolomics biomarkers that contribute to anticipated outcomes thanks to our interpretable model. Early identification of T2DM patients by KTBoost facilitates clinical decision-making and the best use of available resources.

## Data Availability

Data can be requested from the corresponding author upon appropriate request. Requests to access these datasets should be directed to FY, hilal.yagin@inonu.edu.tr.
